# The feasibility of a modified shoe for multi-segment foot motion analysis: a preliminary study

**DOI:** 10.1186/s13047-016-0138-5

**Published:** 2016-02-24

**Authors:** J. Halstead, A. M. Keenan, G. J. Chapman, A. C. Redmond

**Affiliations:** Leeds Institute of Rheumatic and Musculoskeletal Medicine, University of Leeds, 2nd Floor, Chapel Allerton Hospital, Harehills Lane, Leeds, LS7 4SA UK; NIHR Leeds Musculoskeletal Biomedical Research Unit, Leeds Teaching Hospitals Trust, Leeds, UK; Arthritis Research UK Experimental Arthritis Centre, Leeds, UK; Arthritis Research UK Centre for Sports, Exercise and Osteoarthritis, Nottingham, UK; Arthritis Research UK Centre for Sports, Exercise and Osteoarthritis, Oxford, UK; Arthritis Research UK Centre for Sports, Exercise and Osteoarthritis, Loughborough, UK; Arthritis Research UK Centre for Sports, Exercise and Osteoarthritis, Leeds, UK

**Keywords:** Foot, Multi-segment, Kinematics, Shoe, Foot pain

## Abstract

**Background:**

The majority of multi-segment kinematic foot studies have been limited to barefoot conditions, because shod conditions have the potential for confounding surface-mounted markers. The aim of this study was to investigate whether a shoe modified with a webbed upper can accommodate multi-segment foot marker sets without compromising kinematic measurements under barefoot and shod conditions.

**Methods:**

Thirty participants (15 controls and 15 participants with midfoot pain) underwent gait analysis in two conditions; barefoot and wearing a shoe (shod) in a random order. The shod condition employed a modified shoe (rubber plimsoll) with a webbed upper, allowing skin mounted reflective markers to be visualised through slits in the webbed material. Three dimensional foot kinematics were captured using the Oxford multi-segment foot model whilst participants walked at a self-selected speed.

**Results:**

The foot pain group showed greater hindfoot eversion and less hindfoot dorsiflexion than controls in the barefoot condition and these differences were maintained when measured in the shod condition. Differences between the foot pain and control participants were also observed for walking speed in the barefoot and in the shod conditions. No significant differences between foot pain and control groups were demonstrated at the forefoot in either condition.

**Conclusions:**

Subtle differences between pain and control groups, which were found during barefoot walking are retained when wearing the modified shoe. The novel properties of the modified shoe offers a potential solution for the use of passive infrared based motion analysis for shod applications, for instance to investigate the kinematic effect of foot orthoses.

## Background

Examining the biomechanical effect of footwear and in-shoe orthotic devices using multi-segment kinematics can be problematic. A recent review showed the majority of studies using multi-segment foot models, were limited to assessment of barefoot function [1], as shoes confound surface marker placement. Traditional single segment foot models have required a minimum of three skin-mounted markers on the foot and one on the tibia. In-shoe kinematic studies have overcome this by removing sections (windows) of material from the heel counter and shoe upper to accommodate the skin-mounted markers [2, 3]. As modern multi-segment foot models require eight or more skin mounted markers dispersed across the foot, recent studies have employed sandals to accommodate surface reflective markers [4, 5]. This is a particular problem for the evaluation of in-shoe orthoses, which are difficult to secure in sandals and may alter how the insoles perform when measured in an enclosed shoe.

To understand the movement of the foot and its interaction with a shoe, some studies have developed multi-segment foot models using infra-red markers mounted on the skin or on wands that can be visualised by cutting multiple windows into the footwear [6], although given the size of and number of holes (depending on the model), this may alter the structural integrity of the shoe. The presence of a shoe upper has been shown to affect function from heel lift to toe-off, which may be an important factor for in-shoe orthoses studies [7, 8]. In addition, footwear can alter temporal and spatial gait parameters, joint kinematics and kinetics in adults and children [9–11], and it is not clear to what extent this occurs with a sandal compared to a modified closed-toe shoe with apertures for surface markers. The current approach (described herein) minimises this effect by replacing the shoe upper with a webbing material that can provide some features of enclosed footwear while also allowing visualisation of the surface markers. This approach may provide an alternative solution for gait studies that wish to measure in-shoe multi-segment kinematics and also explore the mechanism of action of foot orthoses. The aim of this feasibility study was to investigate the clinical application of a modified shoe (with a webbing upper) intended to accommodate a multi-segment foot marker set without compromising the measurement of foot kinematics using infra-red surface markers. Foot kinematics were firstly compared barefoot and within shoe and, secondly compared between two groups: foot pain and control group.

## Methods

### Study design

**Fig. 1:**
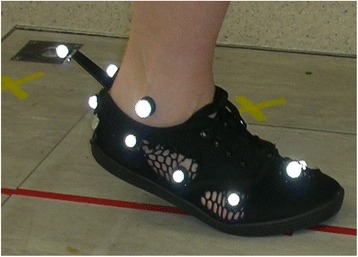
Depicts the webbed upper of the modified shoe during gait analysis with the Oxford multi-segment foot model marker set in situ

This modified shoe (Fig. 1) was assessed in this feasibility study firstly by examining the repeatability of the foot kinematics between the two conditions; barefoot and wearing the modified shoe (hereafter referred to as the shod condition), in the entire participant sample. Secondly a comparison was made between the two groups; foot pain and pain-free control group and, in both conditions (barefoot and shod) to explore whether between group differences in barefoot foot kinematics were maintained while under the shod condition.

### Modification of the shoe

A shoe was modified to accommodate the foot, foot orthoses and allow for multi-segment foot kinematics to be measured in accordance with the Oxford Foot Model [12]. A basic plimsoll style shoe was chosen for modification that had a canvas upper, conventional laced fastening and a flat 6 mm rubber sole unit. This particular shoe was chosen to be modified for gait studies because of its properties; having no specific features that may alter the foot in a similar manner to foot orthoses, such as a raised heel, thick sole (over 7 mm), rigid heel cup or contoured arch, features that have been shown to affect foot kinematics and in-shoe pressures systematically [9, 13]. The upper of the shoe was modified by customising the material rather than removing it, with the canvas panels replaced by a rigid webbing material with similar tensile strain properties. All shoe seams that support the shape and function of the upper were preserved. The webbing material was chosen to enable the protrusion of reflective markers through the mesh to be visible for motion capture during gait analysis, with customised slits added locally to eliminate impeding of markers. Once the design was finalised and the initial testing and optimisation complete, replicas were made in the UK sizes four to 11 (Fig. 1).

### Participants

In accordance with the Declaration of Helsinki, ethical approval was provided by Leeds West Ethics Committee (reference number: 09/H1305/10). We recruited a convenience sample of 15 participants with midfoot pain from local podiatry departments and a control group of 15 pain-free volunteers, recruited from university and hospital staff. Inclusion criteria for foot pain group were; (i) an episode of intermittent medial midfoot pain during weight-bearing activities of over 3 months duration, (ii) the type of pain consistent with pain of mechanical origin as judged by an experienced musculoskeletal specialist podiatrist (JH), (iii) aged 18 or over and able to understand and provide informed consent. Exclusion criteria for the foot pain group were as follows (i) established OA of the midfoot region (confirmed by definite joint space loss or osteophytosis in the radiography report), (ii) foot surgery in the last 12 months, (iii) foot pain typical of undiagnosed inflammatory arthritis (sudden foot pain with diurnal variation or at rest or asleep, early morning stiffness of 30 min or more, multiple inflamed joints, bursitis, tenosynovitis and enthesitis), (iv) foot pain typical of neurological pain (pain at rest, referred pain, allodynia, diffuse pain that it described as burning and or tingling) and any participants presenting with foot pain that is constant, unremitting or nocturnal pain, (v) any medical history of unstable diabetes mellitus or diabetic complications, kidney disease, peripheral arterial disease, systemic inflammatory disease, unstable heart disease and recent heart bypass surgery in the last 6 months, (vi) neurological disorders or sensation loss at the feet (pedal absent vibration or monofilament), an organ transplantation or currently wearing in-shoe orthotic devices.

In the control group, inclusion criteria were; (i) ability to walk for 30 min without pain or discomfort in any lower limb joints, (ii) aged 18 or over and able to understand and provide informed consent. Exclusion criteria for the control group were; (i) a history of foot pain in the last 24 months, (ii) a medical history of: unstable diabetes mellitus and/ or diabetic complications, peripheral arterial disease, systemic inflammatory disease, kidney disease and organ transplantation.

### Data capture

Each participant undertook one session of gait analysis in two conditions, barefoot and shod, in a random order (markers were not removed between conditions). A single foot was analysed; either the more painful limb (foot pain group) or the dominant limb (control group). Multi-segment foot kinematics for the test limb were captured using 9.5 mm reflective markers attached to the skin, by a single clinician (JH) in accordance with the Oxford foot model [12]. The Oxford foot model is a based on the Grood and Suntay joint co-ordinate system [14]. The model was applied according to the definitions proposed by Stebbins et al. for the hindfoot, forefoot and hallux segments [12]. The angles of rotation are defined in the following order for the hindfoot relative to the tibia as follows: plantar/dorsiflexion about the transverse axis of the tibia, inversion/eversion about the longitudinal axis of the hindfoot, internal/external rotation about an axis perpendicular to the previous two axes. The angles of rotation are defined in the following order for the forefoot relative to the hindfoot as follows: plantar/dorsiflexion about the transverse axis of the hindfoot, supination/pronation about the longitudinal axis of the forefoot and abduction/adduction about an axis perpendicular to the previous two axes. The hallux is segment is relative to the forefoot producing a vector with a single axis of rotation: plantar/dorsiflexion about the transverse axis of the forefoot. This model and the Vicon Polygon Oxford foot model processing pipeline has been used and is well described in the literature [15, 16].

Kinematic data was collected at 200Hz using an eight camera motion capture system (Vicon MX, Oxford Metrics, UK), integrated with force plates (Bertec Corporation, USA) capturing at 1000Hz. Participants were then placed in a static reference neutral position (FPI score = 0 [17] with the feet perpendicular, forefoot and hindfoot aligned and the subtalar joint neutral with the talar head palpated equally at the anterior ankle) for each condition as foot kinematics have been shown to be more consistent with a comparable off-set [16, 18]. All participants completed a five minute acclimatisation period wearing the shoes prior to data collection. For each experimental condition, every participant completed eight walks (10 m per walk), at a self-selected walking speed, with gait events, such as heel strike and toe off identified by the force plate and using the built-in auto-correlation function to determine the marker trajectories and map these to the subsequent heel strike and toe-off events, which occur beyond the force plates.

### Data analysis

Kinematic data was filtered using a Woltring fifth-order spline-interpolating function [19] and hindfoot and forefoot kinematics were processed using Vicon Polygon version 3.1. Sagittal and frontal plane motion of the hindfoot relative to the tibia and sagittal and transverse plane motion of the forefoot relative to the hindfoot were specifically chosen for further analysis, as these were shown to be reliable and most clinically relevant as demonstrated when examining differences between participants with normal and flat feet, also known as pes planus [15, 20]. Data was assessed graphically and analysed using SPSS version 19 (IBM, USA) with the significance level set at *p* = <0.05. Trials for each participant were normalized to 100 % of the gait cycle and collectively averaged at 2 % intervals to produce 51 data points. For each participant, hindfoot and forefoot segmental plots for all eight trials were plotted in the pre-selected planes. The single most representative gait cycle was chosen for further analysis by a single researcher (JH) using Polygon version 3.1 reporting template. Motion-time consistency plots for all eight gait cycles were produced in the same graph and the trial that was closest to the middle of the plots for most of the stance duration was chosen as the most representative for each plane per person. This approach was chosen over a mean of multiple trials to ensure peak values were maintained as part of the natural variability, rather reducing peak motions in order to enhance reliability [21, 22].

Due to the feasibility nature of the present study, a conservative statistical analysis approach was employed. The reliability of the kinematic graphical outputs between the experimental conditions: barefoot and shod, was examined across the entire sample (combining both groups, *n* = 30) to include a wide range of values using a coefficient of multiple correlation (CMC) as described by Kadaba et al. [23]. The results were described as poor, fair-to-good, and excellent reliability and assigned cut-off values of <0.40, between 0.40 and 0.75, and >0.75, respectively [20, 24]. Due to the small sample size recruited in this feasibility study, no attempt was made to examine the reliability between barefoot and shod trials within groups. Due to the small sample size and heterogeneous distribution of the continuous data, non-parametric tests for differences were employed. To compare the walking speed and kinematic differences between groups (foot pain and control) in the both experimental conditions (barefoot and shod), the median value and inter-quartile range (IQR) was compared using Mann–Whitney-U tests. For the kinematic data, 50 % of stance (mid-stance) was chosen as a time point to compare data in order to minimise the effect of skin artefact on marker distances [25, 26] and also to represent the time in the gait cycle when the mechanism of in-shoe orthotic devices are proposed to work, particularly in a group with midfoot pain [27].

## Results

Foot pain participants were matched for gender (11 females in each group) and with-in 5 years for age, which resulted in an older median age of 56 years (range 22 to 76 years) in the foot pain group compared to the control group who had a median age of 49 years (range 27 to 72 years). Both groups were of similar height, median 1.62 m (range 1.54 m to 1.78 m) in the control group, and median 1.66 m (range 1.50 m to 1.80 m) in the foot pain group. Body mass index (BMI) was slightly greater in the foot pain group (median BMI = 28.7; range 23.6 to 43.5) compared to the control group (median BMI = 26.7; range 19.9 to 29.2).

The repeatability between barefoot and shod kinematics was good to excellent in the whole group (combined sample of foot pain and control groups, *n* = 30). The mean hindfoot sagittal plane and mean frontal plane motions were the most repeatable, with an excellent CMC of 0.967, and 0.981, respectively. Forefoot sagittal plane motions showed good agreement (CMC = 0.743). While forefoot transverse plane motions across the group were graphically similar, there was a mean offset of four degrees in the absolute values, which prevents computation of the CMC. After correction for this offset (as described by Kadaba et al. [23]), the CMC for forefoot transverse plane motion was excellent 0.899.

The comparison between groups suggests the foot pain group walked significantly slower (barefoot: median 1.07 m/s, IQR 0.22 m/s; shod: 1.08 m/s, IQR 0.23 m/s) compared to the control group (barefoot: median 1.23 m/s, IQR 0.19 m/s; shod: 1.22 m/s, IQR 0.26 m/s) under both conditions; barefoot (median difference 0.16 m/s *p* = 0.000) and shod (median difference 0.14 m/s *p* = 0.023).

**Fig. 2:**
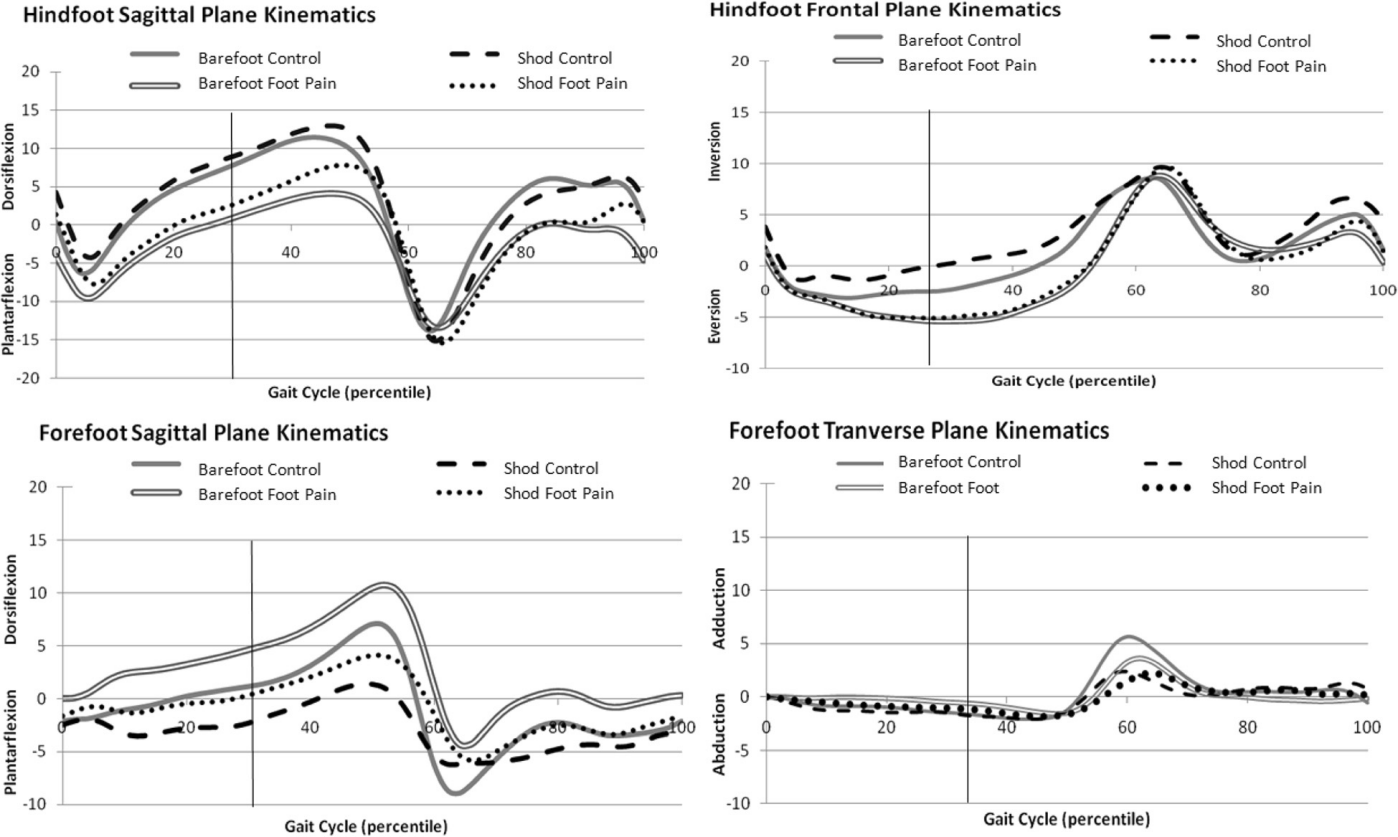
Foot kinematic plots for both groups under both conditions for pre-determined planes and variables of choice. The figure illustrates hindfoot sagittal (hindfoot dorsiflexion is positive) and frontal planes (hindfoot inversion is positive) kinematics as well as sagittal (forefoot dorsiflexion is positive) and transverse plane (forefoot adduction is positive) forefoot kinematics. Grey single and black dashed lines represent the control group under barefoot and shod conditions, respectively. Grey double and black dotted lines represents the foot pain group under barefoot and shod conditions, respectively. The vertical line in all graphs represents 50 % of stance.

Figure 2 illustrates the mean kinematic time series plots for each group in both conditions. For statistical correctness in the subsequent inferential testing Table 1 outlines the median differences between the foot pain and control groups and differences across the two conditions; barefoot and shod at 50 % of the stance phase. Note therefore that direct comparison should not be made between the figure and the data in the table.

**Table 1:** Between group differences for pre-determined foot kinematics at 50 % of stance (median and inter-quartile range [IQR]) are expressed in both conditions

Median Values (50 % Stance)	Hindfoot Sagittal		Hindfoot Frontal		Forefoot Sagittal		Forefoot Transverse	
Barefoot		IQR		IQR		IQR		IQR
Control	9.62°	8.72°	−3.18°	7.39°	1.95°	7.61°	−1.59°	2.87°
Barefoot		IQR		IQR		IQR		IQR
Foot Pain	2.00°	4.93°	−4.45°	7.19°	5.82°	8.80°	−0.03°	2.49°
Difference	7.62°	U = 46.5, *p* = 0.005	1.27°	U = 69.5, *p* = 0.074	−3.87°	U = 67.5, *p* = 0.061	−1.56°	U = 70.5, *p* = 0.081
Shod		IQR		IQR		IQR		IQR
Control	12.48°	10.06°	−0.86°	6.34°	−0.88°	2.94°	−2.32°	2.25°
Shod		IQR		IQR		IQR		IQR
Foot Pain	4.02°	10.20°	−4.66°	5.37°	1.78°	9.74°	−1.46°	2.05°
Difference	8.46°	U = 51, *p* = 0.01	3.81°	U = 39, *p* = 0.002	−2.66°	U = 91, *p *= 0.389	−0.86°	U = 86, *p* = 0.285

Hindfoot dorsiflexion is positive, Hindfoot inversion is positive, Forefoot dorsiflexion is positive, Forefoot adduction is positive

### Hindfoot kinematics

Under both the barefoot and shod conditions, the foot pain group demonstrated significantly less hindfoot dorsiflexion compared to the control group (barefoot difference 7.6°, *p* =0.005; shod difference 8.5°, *p* = 0.01). Despite these findings, the shod condition had minimal effect on hindfoot kinematics compared to the barefoot condition for both groups.

There was a trend toward the foot pain group demonstrating greater hindfoot eversion during barefoot walking (median eversion = −4.5°, IQR 7.2°) compared to the control group (median eversion = −3.2°, IQR 7.4°), but this was not statistically significant (*p* = 0.074). Under the shod condition, however, the foot pain group demonstrated significantly greater hindfoot eversion (median eversion = −4.7°, IQR 5.4°) compared to the control group (median eversion = −0.9°, IQR 6.3°; *p* = 0.002).

### Forefoot kinematics

There were no significant differences in sagittal or transverse plane forefoot kinematics between groups for either condition (see Table 1).

## Discussion

The present study aimed to investigate the feasibility of using a modified shoe with a webbed upper in order to accommodate and measure multi-segment in-shoe foot kinematics of control and foot pain participants without compromising foot kinematics between barefoot and shod conditions. The current findings demonstrated that under the barefoot condition, there were differences in foot kinematics between the foot pain group and the control group and, measurement of these differences could be maintained under shod conditions, suggesting that this modified shoe had minimal effect on foot kinematics.

The repeatability of walking barefoot and in the modified shoe was excellent for three of the four pre-determined foot kinematic measures (hindfoot sagittal, hindfoot frontal and forefoot transverse) and good for the fourth measure (forefoot sagittal). This compares well to previous repeatability data in a group of patients with rheumatoid arthritis [20, 28], although the forefoot sagittal repeatability was lower in this study, potentially due to separate within-day measures barefoot and shod. Our findings suggest that the modified shoe had little effect on foot kinematics when compared to barefoot measurements. Further between-day repeatability studies are recommended for application in prospective studies.

In previous biomechanical studies, removing sections of material from the upper of shoes has been undertaken to examine the effect of footwear on multi-segment foot kinematics [8, 29]. The optimum size of the material removal in trainers has shown to be 25 mm, which can improve tracking using markers mounted on wands (visualised outside of the shoe) and maintain shoe integrity [8, 30]. This may, however have limited application due to the number and position of the holes needed for direct surface markers (depending on the model used) potentially requiring customisation for different foot shapes and postures so that each participant would require an individualised shoe. Movement of the foot within the shoe may obscure surface marker(s), which may limit the choice of multi-segment foot models to one using markers mounted on wands. Our solution, to replace the shoe upper with a webbed material rather than to remove all material allowed visualisation of the bony landmarks and skin mounted markers while preserving more of the shoe structure. This modified shoe has potential applications for gait studies that aim to test the mechanism of action of orthoses with specific characteristics, which is recommended for phase one explorative device studies [31] as it has minimal features that may potentially influence the foot in a similar fashion to foot orthoses. It is recognised, however that this shoe is not representative of sports trainers, work or office footwear and that further research to investigate multi-segment kinematics in a wide range of footwear would be beneficial to understand the interactions with the shoe, foot and in-shoe orthotic devices.

Under barefoot conditions, our results demonstrate that the foot pain group walked significantly slower than the control group. This is unsurprising, considering that our patient group reported pain during weight-bearing activities of daily living. Similarly, the foot pain group also demonstrated significantly less hindfoot dorsiflexion and a trend towards a greater hindfoot eversion compared to the control group. It should be noted that especially for non-significant results, where we have indicated a trend towards an effect, the small differences in measures such as hindfoot eversion may lie within the measurement error [32, 33]. These observed differences in walking speed and foot kinematics were still maintained under shod conditions. These kinematic results are similar to another comparative study using the same Oxford foot model in a sample with a pes planus deformity [15]. The results of this and the aforementioned study would suggest that altered kinematics in people with foot pain and foot deformity can be targeted with in-shoe orthotic devices.

At present, there are very few orthotic device studies using multi-segment foot models [34–36], mainly due to the difficulties in accommodating multiple surface markers in-shoe and the requirement for removal of large amounts of material from the shoe upper. The mechanism of action of foot and/or ankle orthoses either in experimental studies or clinical studies can only be examined in-shoe. To standardise the interaction of foot orthoses within footwear, it is important to modify a shoe appropriately to maintain the properties of the shoe whilst being able to determine how the orthotic device works using biomechanical analyses.

Our solution to modify the upper of the shoe to visualise the skin-mounted markers was a novel solution to the measurement of in-shoe kinematics. With our data demonstrating good to excellent between-condition repeatability, combined with maintaining observed differences in foot kinematics between foot pain and control participants, suggests the modified shoe with a webbed upper is a potential solution for future studies examining in-shoe foot kinematics. The shoe also has the potential for orthoses studies as the inlay of the plimsoll shoe was removable, allowing for the fitting of foot orthoses up to a 6 mm heel depth. Future research is required to determine the between day and within day reliability of in-shoe foot kinematics when wearing this modified shoe, both in a range of participants with different foot postures and when examining the effect of different foot orthoses with variable depth.

Kinematics of the forefoot segment in barefoot and shod conditions were variable (see Table 1 and Fig. 2). Forefoot variability shown in this study may possible due to skin artefact errors that are known to be highest for the forefoot segment [37]. In addition measuring the forefoot as an entire segment, as defined in Oxford model, has shown greater variability than separating the metatarsals for instance into medial and lateral forefoot segments as shown in later foot models [38]. Further studies examining the effect of a modified shoe using a different multi-segment model would be recommended.

The modified shoe did not have a significant effect on walking speed in the control group, which suggests a minimal effect on healthy walking function, This was in agreement with previous studies, which have shown minimal changes in gait parameters when wearing soft flexible footwear in groups of adults and children without foot pain [9, 39]. The modified shoe also had no significant effect on walking speed in patients with foot pain, the differences between the foot pain and control groups was maintained. In contrast, gait speed has been shown to be altered when wearing footwear in people with severe pain and deformity associated with rheumatoid arthritis [28], perhaps due to the effect of the shoe to mediate of foot pressures [40, 41]. This would suggest the application of a modified shoe to measure in-shoe kinematics in a group of people with painful deformity would require further study.

The results of this study should be considered within the in the context of the following limitations. The forefoot segment kinematic outputs demonstrated high between-subject variability in both barefoot and in shod conditions, however this is consistent with similar studies [1, 16, 42]. This and other studies using a single forefoot segment confirm therefore that forefoot motions, whether obtained barefoot or shod, require cautious interpretation. The off-set of the kinematic variables shown in the forefoot transverse plane may have reflected positional differences of the foot within shoe, however this off-set was relatively low compared to previous OFM data collected using the same software and equipment suggesting comparable data [12]. A final limitation in this study is the choice of single representative trial rather than a mean of multiple trials. The influence of trial number has not been fully explored in walking foot kinematics however, data from running trials of single versus multiple trials can provide small increases in reliability [21].

## Conclusion

The present study demonstrated that individuals with foot pain had significantly different hindfoot kinematics to those of control participants when walking under barefoot conditions. Importantly, some of these observed differences were preserved when wearing the modified shoe. The present study suggests that modifying a shoe with webbed upper is a practical solution to allow accurate measurement of multi-segment foot kinematics using surface-mounted passive infra-red marker sets under shod conditions.
